# Integrative radiogenomics analysis for predicting molecular features and survival in clear cell renal cell carcinoma

**DOI:** 10.18632/aging.202752

**Published:** 2021-03-26

**Authors:** Hao Zeng, Linyan Chen, Manni Wang, Yuling Luo, Yeqian Huang, Xuelei Ma

**Affiliations:** 1 Department of Biotherapy, Cancer Center, State Key Laboratory of Biotherapy, West China Hospital, Sichuan University, and Collaborative Innovation Center, Chengdu, China; 2 West China School of Medicine, West China Hospital, Sichuan University, Chengdu, China

**Keywords:** renal cell carcinoma, radiomics, genomics, transcriptomics, proteomics

## Abstract

Objectives: To assess the feasibility of predicting molecular characteristics by computed tomography (CT) radiomics features, and predicting overall survival (OS) using combination of omics data in clear cell renal cell carcinoma (ccRCC).

Methods: Genetic data of 207 ccRCC patients was retrieved from The Cancer Genome Atlas (TCGA) and matched contrast-enhanced CT images were obtained from The Cancer Imaging Archive (TCIA). Another cohort of 175 ccRCC patients from West China Hospital was used as external validation. We first applied radiomics features and machine learning algorithms to predict genetic mutations and mRNA-based molecular subtypes. Next, we established predictive models for OS based on single omics, combined omics (radiomics+genomics, radiomics+transcriptomics, radiomics+proteomics) and all features (multi-omics).

Results: Using radiomics features, random forest algorithm showed good capacity to identify the mutations *VHL* (AUC=0.971), *BAP1* (AUC=0.955), *PBRM1* (AUC=0.972), *SETD2* (AUC=0.949), and molecular subtypes m1 (AUC=0.973), m2 (AUC=0.968), m3 (AUC=0.961), m4 (AUC=0.953). The TCGA cohort was divided into training (n=104) and validation (n=103) sets. The radiomics model had promising prognostic value for OS in validation set (5-year AUC=0.775) and external validation set (5-year AUC=0.755). In the validation set, the radiomics+omics models enhanced predictive accuracy than single-omics models, and the multi-omics model made further improvement (5-year AUC=0.846). High-risk group of validation set predicted by multi-omics model showed significantly poorer OS (HR=6.20, 95%CI: 3.19-8.44, p<0.0001).

Conclusions: CT radiomics might be a feasible approach to predict genetic mutations, molecular subtypes and OS in ccRCC patients. Integrative analysis of radiogenomics may improve the survival prediction of ccRCC patients.

## INTRODUCTION

Renal cell carcinoma (RCC) is a diverse group of carcinomas including three common histopathological subtypes (clear cell, papillary, chromophobe) and other rare subtypes, which resulted in 403,300 new cases and 175,100 deaths worldwide [1, 2]. Clear cell renal cell carcinoma (ccRCC) is the most prevalent type (70-75%), and more prone to advanced T stage, higher nuclear grade, metastatic lesions and poorer prognosis than papillary and chromophobe RCC [3, 4]. Patient status, tumor stage, nuclear grade, coagulative necrosis and proinflammatory markers are commonly used prognostic indicators in clinical practice [5]. However, precise treatments of ccRCC are developing rapidly, thus it is necessary to continuously evaluate the emerging prognostic factors to enable clinicians to individually risk-stratify and select the optimal therapeutic strategy for patients.

Recent advances in genetics performed extensive genomics and transcriptomics profiling to reveal the underlying molecular mechanism of ccRCC [6]. The characteristic of ccRCC is the loss of chromosome 3p, including mutations of genes encoding von Hippel-Lindau tumor suppressor (*VHL*), polybromo-1 (*PBRM1*), *BRCA1*-associated protein 1 (*BAP1*) and SET domain containing 2 (*SETD2*) [6]. The VHL pathway is essential for adaptation to hypoxia. Loss of VHL function and dysregulated hypoxia lead to the activation of downstream pathways associated with angiogenesis [7]. Several targeted blockers of vascular endothelial growth factor pathway (e.g., sorafenib, axitinib) have been approved for treatment of metastatic RCC [8]. However, the capability of *VHL* mutation as a prognostic factor was not significant in ccRCC [9]. Conversely, *PBRM1* mutation was associated with shorter survival, early tumor progression, and response to immune checkpoint therapies in ccRCC patients [10, 11]. Patients with *BAP1* or *SETD2* mutation were also more likely to have worse prognosis [12], and *BAP1* mutation was related with poor response to everolimus [13]. Furthermore, the four molecular subtypes obtained from gene expression pattern analysis showed different genetic alterations and survival probabilities [6]. Taken together, these molecular features have implications to prognosis prediction and treatment strategies for ccRCC patients. Given the invasive and high-cost molecular assays, developing non-invasive and cost-effective biomarkers for these mutations and molecular subtypes would be important.

Quantitative image analysis can capture distinct imaging phenotypes that cannot be recognized by naked eye, and reveal the underlying pathophysiology of biomedical images [14]. Radiomics is the practice of converting digital images into mineable quantitative features, then performing subsequent analyses to improve accuracy of differentiating tumor types and grade, predicting prognosis and therapeutic response [15]. Radiogenomics refers to the investigation of connection between radiomics and genomics data, which has extended to link radiomics to broader biological features such as proteomics and metabonomics in recent years [16]. Previous studies have used radiomics features to non-invasively identify gene expression, mutations, molecular subtypes and methylation status within the tumors [17–20]. In addition, the integration of radiomics and omics has been reported to achieve a more accurate prediction of survival in several cancers [21–23]. Therefore, the radiogenomics analysis might offer insights into the molecular phenotypes of cancer, and provide predictive biomarkers for personalized management of cancer patients.

In ccRCC, computed tomography (CT) is routinely used to capture the physical characteristics of the whole tumor volume. Radiomics features extracted from CT scans have showed significant ability to predict mutation status of *VHL*, *BAP1* and *PBRM1* in ccRCC [24, 25]. To our knowledge, no study has applied CT image features to identify molecular subtypes, and performed multi-omics analysis to predict prognosis of ccRCC patients. Therefore, we first aimed to comprehensively evaluate the potential value of CT radiomics features in classifying mutations and molecular subtypes of ccRCC, with the use of multiple machine learning algorithms. Next, the present study established and validated various predictive models (radiomics, genomics, transcriptomics, proteomics, multi-omics) to enhance the prediction of overall survival in patients with ccRCC.

## RESULTS

### Predicting mutations and molecular subtypes from radiomics features

**Figure 1:**
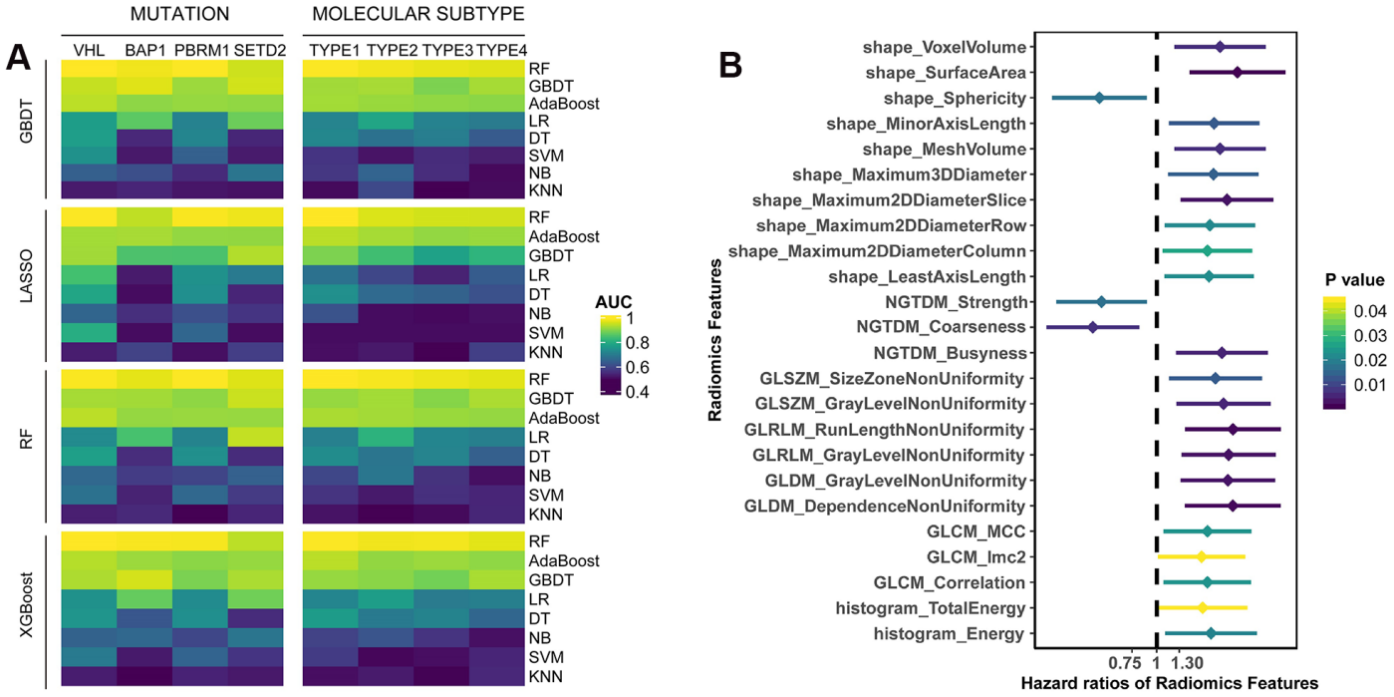
(**A**) The predictive performance of radiomics features for somatic mutations and molecular subtypes in test set. Four algorithms (GBDT, LASSO, RF, XGBoost) were used for feature selection, and eight algorithms (RF, GBDT, AdaBoost, LR, DT, SVM, NB, KNN) were utilized for classification. (**B**) Univariate survival analysis of radiomics features. Patients were divided into two groups based on the median value of each feature.

In this study, we included 207 ccRCC patients from TCGA dataset (Table 1). To present the clinical utility of radiomics features in ccRCC, we first estimated the power of radiomics features to predict four commonly mutated genes (*VHL*, *BAP1*, *PBRM1* and *SETD2*) of 137 patients and four molecular subtypes (m1-m4) reflected by mRNA patterns of 180 patients (Table 1). We used four algorithms (GBDT, LASSO, RF, XGBoost) for feature selection, and eight algorithms (RF, GBDT, AdaBoost, LR, DT, SVM, NB, KNN) as classifiers. Among the 32 combinations of two algorithms, the RF achieved the best performances on the test set no matter which feature selection method was adopted (Figure 1A). The radiomics models based on RF were able to distinguish the mutations *VHL* (AUC=0.971), *BAP1* (AUC=0.955), *PBRM1* (AUC=0.972), *SETD2* (AUC=0.949), and subtypes m1 (AUC=0.973), m2 (AUC=0.968), m3 (AUC=0.961), m4 (AUC=0.953) in the test set (Supplementary Table 1). The models built by GBDT and AdaBoost also obtained comparable accuracy to that of RF model, suggesting that radiomics features were capable of identifying somatic mutations and molecular subtypes of ccRCC through machine learning.

**Table 1:** Demographic and clinical characteristics of patients.

**Characteristic**	**TCGA**			**p value^1^**	**External validation set**	**p value^2^**
	**Total**	**Training set**	**Validation set**			
No. of patients	207	104	103	-	175	-
Age: mean ± SD	59.8 ± 12.2	60.1 ± 12.6	59.5 ± 11.9	0.695	56.3 ± 12.6	0.007
Overall survival (mo)	45.5 ± 30.8	46.6 ± 31.4	44.3 ± 30.3	0.597	45.8 ± 30.5	0.923
Gender						
Male	135 (65.2%)	64 (61.5%)	71 (68.9%)		108 (61.7%)	
Female	72 (34.8%)	40 (38.5%)	32 (31.1%)	0.264	67 (38.3%)	0.478
TNM stage						
I	107 (51.7%)	54 (51.9%)	53 (51.5%)		104 (59.4%)	
II	18 (8.7%)	13 (12.5%)	5 (4.9%)		24 (13.7%)	
III	51 (24.6%)	20 (19.2%)	31 (30.1%)		29 (16.6%)	
IV	31 (15.0%)	17 (16.3%)	14 (13.6%)	0.101	18 (10.3%)	0.051
Tumor grade						
G1-G2	82 (39.6%)	39 (37.5%)	43 (41.7%)		124 (70.9%)	
G3-G4	125 (60.4%)	65 (62.5%)	60 (58.3%)	0.532	51 (29.1%)	<0.001
VHL mutation						
No	64 (46.7%)	29 (40.8%)	35 (53.0%)		NA	
Yes	73 (53.3%)	42 (59.2%)	31 (47.0%)	0.153		-
BAP1 mutation						
No	123 (89.8%)	66 (93.0%)	57 (86.4%)		NA	
Yes	14 (10.2%)	5 (7.0%)	9 (13.6%)	0.203		-
PBRM1 mutation						
No	81 (59.1%)	41 (57.7%)	40 (60.6%)		NA	
Yes	56 (40.9%)	30 (42.3%)	26 (39.4%)	0.835		-
SETD2 mutation						
No	127 (92.7%)	66 (93.0%)	61 (92.4%)		NA	
Yes	10 (7.3%)	5 (7.0%)	5 (7.6%)	0.734		-
Molecular subtypes						
m1	58 (32.2%)	31 (34.1%)	27 (30.3%)		NA	
m2	41 (22.8%)	25 (27.5%)	16 (18.0%)			
m3	48 (26.7%)	16 (17.6%)	32 (36.0%)			
m4	33 (18.3%)	19 (20.9%)	14 (15.7%)	0.040		-

^1^P value for comparison between training set and test set.

^2^P value for comparison between TCGA total and external validation set.

### Prognostic value assessment of radiomics features

According to median value of radiomics features, the patients were divided into two groups (higher than median vs. lower than median). Univariate Cox analyses showed that 24 features were significantly associated with overall survival (OS) (Supplementary Table 2), most of which were adverse prognostic factors (p<0.05, Figure 1B). Moreover, we performed multivariate LASSO-Cox regression analysis, and selected four features with non-zero coefficients, including sphericity, surface-to-volume ratio, GLCM_correlation and GLDM_gray-level non-uniformity (GLNU). Kaplan-Meier curves showed the survival differences between groups with high-value or low-value features (Supplementary Figure 1). Except surface-to-volume ratio, other three features were significantly predictive of OS. Taken together, it indicated that individual radiomics features had the potential to predict OS of ccRCC patients.

### Integrating radiomics with genomics features to predict survival

**Figure 2:**
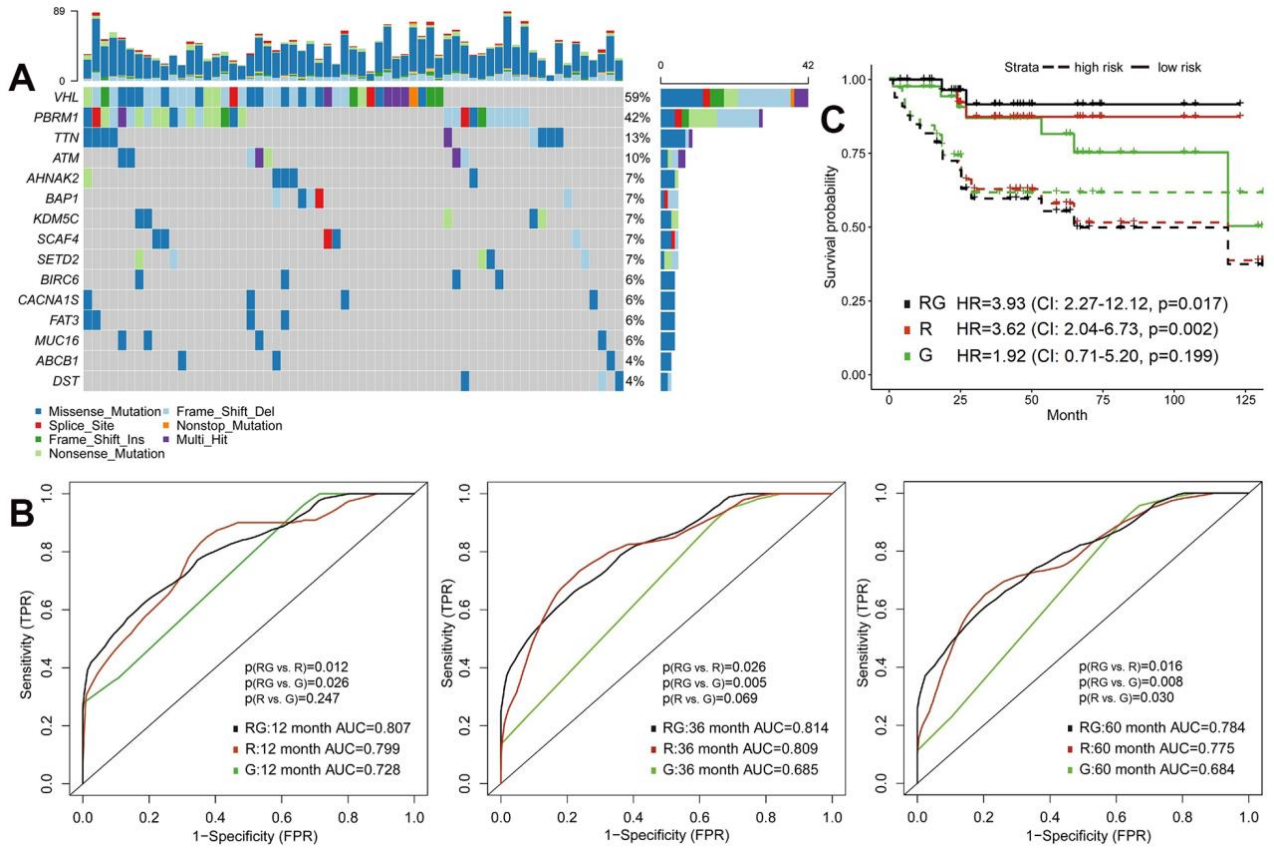
**Predictive models of survival integrating radiomics with genomics features.** (**A**) The waterfall plot of 20 most common somatic mutations in training set. (**B**) The 1-year, 3-year and 5-year area under the ROC of radiomics model (R), genomics model (G) and radiomics+genomics model (RG) in validation set. (**C**) Kaplan-Meier curves showed survival differences between high-risk and low-risk patients of validation set.

Previous studies have found that mutation status has an important impact on the prognosis of cancer patients. Therefore, we compared the prognostic role of radiomics and genomics features, and combined two omics, hoping to provide more accurate stratification of patients’ prognosis. We randomly divided the TCGA cohort into training (n=104) or validation (n=103) sets (Table 1). The 100 most common somatic mutations of training set were used for models (Supplementary Table 3 and Figure 2A). In the training set, we established models to predict OS based on 107 radiomics features and 100 mutations. Time-dependent ROC is more suitable for time-to-event results than classical ROC, thus we used it to obtain dynamic AUC values throughout these models in the validation set [26].

In the validation set, the AUC of radiomics model (R) were significantly higher than genomics model (G) at 5-year (0.775 vs. 0.684, p=0.030) time point (Figure 2B and Supplementary Table 4). Integrative model of radiomics and genomics (RG) achieved better predictive performance, the 1-year, 3-year and 5-year AUCs of which were 0.807, 0.814 and 0.784. The increase in AUC was obvious compared with radiomics and genomics models (all p<0.05; Figure 2B). We then defined high-risk and low-risk groups by median risk score calculated from each model and drew the Kaplan-Meier curves (Figure 2C). The high-risk groups in radiomics model (HR=3.62, 95%CI: 2.04-6.73, p=0.002) and radiomics+genomics model (HR=3.93, 95%CI: 2.27-12.12, p=0.017) were significantly related with poorer survival.

### Integrating radiomics with transcriptomics features to predict survival

**Figure 3:**
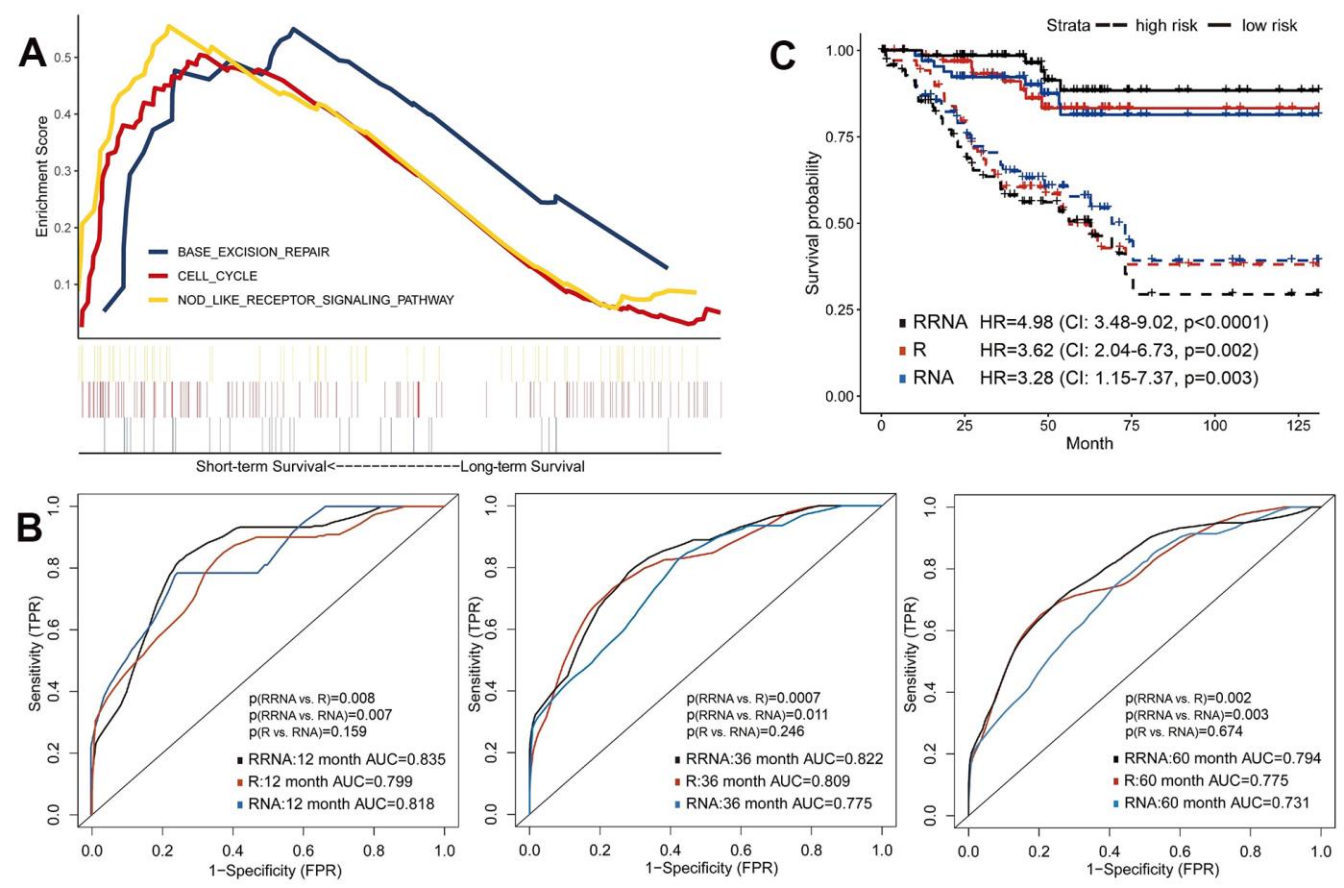
**Predictive models of survival integrating radiomics with transcriptomics features.** (**A**) Gene Set Enrichment Analyses showed three representative pathways enriched in short-term survivors of training set. (**B**) Predictive power of models using radiomics (R), transcriptomics (RNA) or integration of radiomics and transcriptomics (RRNA) in validation set. (**C**) Kaplan-Meier curves of validation set stratified by these models.

Similarly, we selected a part of whole expressed genes to reduce the dimensionality. Patients of training set were assigned into short-term (deceased, 12 months≥OS≥1 month) and long-term (OS≥60 months) survivors. We used the GSEA to reveal enriched KEGG pathways in the short-term survival group (Figure 3A). NOD-like receptors (NLRs) are closely related to autoimmune and inflammatory responses, and chronic inflammation caused by abnormal NLR signaling can promote tumorigenesis and progression, especially in gastric and colorectal cancers [27]. However, the role of NLR pathway in renal cancer remains unclear and needs further investigation.

The 100 most significant DEGs were included in models (Supplementary Table 5). Using the validation set, transcriptomics model (RNA) showed good discrimination ability for OS (1-year AUC=0.818, 3-year AUC=0.775 and 5-year AUC=0.731), which were about equal to the radiomics model (all p>0.05; Figure 3B). Next, the radiomics+transcriptomics model (RRNA) enhanced 1-year AUC to 0.835, 3-year AUC to 0.822 and 5-year AUC to 0.794. Kaplan-Meier analyses demonstrated significantly different survival results between high-risk and low-risk patients (Figure 3C), especially in the integrative model (HR=4.98, 95%CI: 3.48-9.02, p<0.0001).

### Integrating radiomics with proteomics features to predict survival

**Figure 4:**
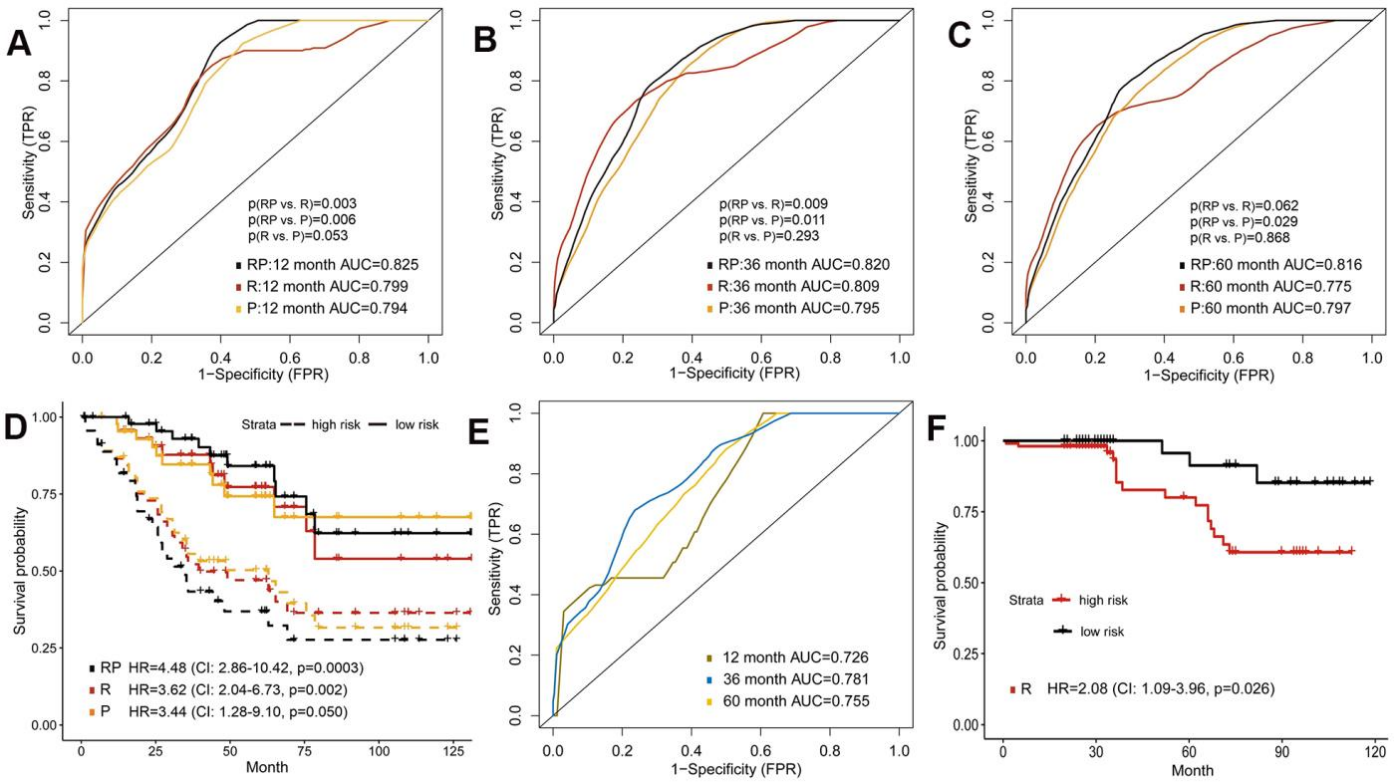
**Predictive models of survival integrating radiomics with proteomics features.** (**A**–**C**) Area under the ROC of radiomics features (R), protein expression (P) and combination of radiomics and proteomics (RP) for predicting survival in validation set. (**D**) Kaplan-Meier curves of the validation set that predicted patients’ survival by above models. (**E**) Predictive ability of radiomics model in external validation set. (**F**) Kaplan-Meier curves of external validation set analyzed with the radiomics model.

The protein expression data was also considered in this study, expecting to improve prognosis prediction. We performed the reverse phase protein array (RPPA) analysis, and included all 131 proteins in the models (Supplementary Table 6). In the validation set, the prediction accuracy of proteomics model (P) was similar to that of radiomics model (all p>0.05; Figure 4A–4C), with 1-year, 3-year and 5-year AUCs of 0.794, 0.795 and 0.797, respectively. Combination of radiomics and proteomics features (RP) yielded highest 1-year AUC of 0.820, 3-year AUC of 0.822 and 5-year AUC of 0.816 (Figure 4A–4C). Furthermore, survival analyses showed that the radiomics+proteomics model outperformed than single-omics models in predicting survival (HR=4.48, 95%CI: 2.86-10.42, p=0.0003; Figure 4D).

### External validation of radiomics model to predict survival

We additionally included 175 ccRCC patients from West China Hospital to verify the prognostic performance of radiomics model. The age of onset was younger and the rate of high-grade tumor was lower in the external validation set (Table 1). However, the radiomics model also successfully predicted patient survival in external validation set, which reached AUCs of 0.726, 0.781, 0.755 for 1-year, 3-year and 5-year OS, respectively (Figure 4E). In Kaplan-Meier survival curves, the high-risk group predicted by radiomics model showed significantly shorter survival time (HR=2.08, 95%CI: 1.09-3.96, p=0.026; Figure 4F).

### Integrating multi-omics features to predict survival

**Figure 5:**
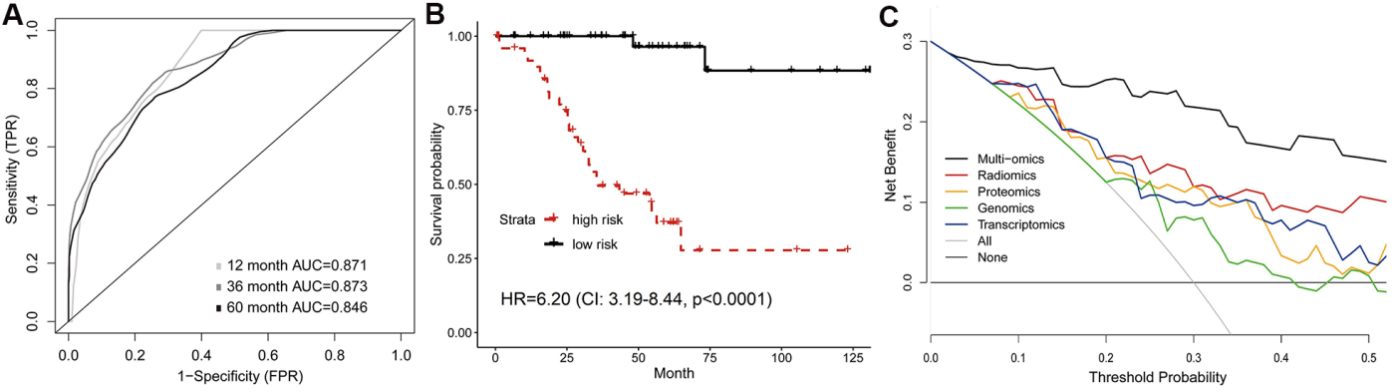
**Predictive model of survival integrating multiple omics features.** (**A**) Time-dependent ROC, and (**B**) Kaplan-Meier curves analyzed by multi-omics model in validation set. (**C**) Comparison of decision curves of each model. The gray oblique line represented the net benefit of intervention for all patients, while horizontal line represented the net benefit of no intervention. The multi-omics model reached higher net benefit than single-omics models across the major range of threshold probability.

Previous results suggested the feasibility of integrating radiomics with genomics, transcriptomics or proteomics to improve survival prediction. Finally, we investigated whether incorporation of all features (radiomics, genomics, transcriptomics and proteomics) could make further improvement on the prognostic model. Tested by the validation set, the 1-year, 3-year and 5-year AUC of the multi-omics model were 0.871, 0.873 and 0.846 (Figure 5A), which were significantly increased compared to the models formed by single omics (Supplementary Table 4). We also found a significant difference between high-risk and low-risk groups’ survival (HR=6.20, 95%CI: 3.19-8.44, p<0.0001; Figure 5B). In addition, DCA curves demonstrated that the multi-omics model added more net benefit than radiomics and other single-omics models when used to predict survival (Figure 5C).

## DISCUSSION

Radiomics analysis offers quantitative image features that can be correlated with clinical or molecular characteristics of cancer [28]. In this study, we mined radiomics features from contrast-enhanced CT, and built various machine learning classifiers based on radiomics features to predict different somatic mutations and molecular subtypes of ccRCC. In addition, we presented a comprehensive framework to evaluate the prognostic value of individual radiomics features and omics-based predictive models. The results demonstrated that the mutations, molecular subtypes and prognosis of ccRCC patients could be predicted from CT radiomics features. Furthermore, the integrative models combining radiomics with gene mutation, gene expression and protein expression features improved the predictive power of overall survival than models constructed by any omics alone. The median risk scores generated by models were feasible to separate patients into high-risk and low-risk groups with significantly different survival outcomes.

As a hallmark of cancer, genetic instability and mutations can lead to uncontrolled cell proliferation [29]. Genomics profiles are widely used as biomarkers to predict survival and therapeutic response, helping to make medical decision, especially the targeted therapy selection [30]. Considering the invasive procedures of genomics testing, radiomics is rapidly emerging as a translational approach to non-invasively identifying mutations and expression patterns [28]. In this context, many efforts have been made to improve radiogenomics analysis in ccRCC [16]. For example, random forest classifier of CT radiomics features correctly identified mutations in *PBRM1* (AUC=0.987) and *BAP1* (AUC=0.897) of ccRCC [31, 32]. In this study, we presented a more comprehensive assessment including four common mutations and 32 combinations of machine learning algorithms. Meanwhile, we applied radiomics features to predict mRNA-based molecular subtypes (m1-m4) of ccRCC, which has not been reported before. Clustering of mRNA expression provides a molecular classification of individual tumors, which facilitates the understanding of underlying tumor heterogeneity [33]. For instance, m1 subtype tumors had significant survival advantages and more frequent *PBRM1* mutation than m2 and m3, while m3 subtype was enriched for *CDKN2A* deletion and *PTEN* mutation [6]. The m4 subtype was related with higher mutation frequency of *BAP1* and *mTOR* [6]. Our results showed that CT radiomics features could be used to develop a non-invasive, convenient and effective tool to predict mutations and molecular subtypes of ccRCC, and random forest algorithm performed best among the eight classifiers.

Timely and accurate identification of patients with high-risk of developing worse outcomes is crucial for their clinical decision-making. For renal cancer, radiomics has been widely applied to predict patient survival and cancer progression [34]. In this study, several radiomics features had prognostic value for OS, including sphericity, GLCM_correlation and GLDM_GLNU. Sphericity (regularity of shape) is a shape-based feature, GLCM_correlation reflects the linear dependency of gray-levels, and GLDM_GLNU describes the non-uniformity of gray-levels of texture [35]. Previous studies also reported that sphericity and GLCM_correlation were associated with survival outcomes in cancer patients [36, 37]. However, evaluation of individual radiomics features was not enough to reflect the whole tumor properties. Therefore, we imputed all radiomics features into the random forest classifier to build a radiomics model, which obtained good predictive capability for OS in both internal validation (5-year AUC=0.775) and external validation sets (5-year AUC=0.755).

Furthermore, we comprehensively estimated the predictive models integrating radiomics with genomics, transcriptomics and proteomics in ccRCC. The results demonstrated that the predictive accuracy of models using two omics was higher than that of single-omics models, and the multi-omics model reached highest accuracy and clinical net benefit. Study for glioblastoma also showed the enhanced predictive capacity of models combining genomics and radiomics features [21]. Radiomics offers important strengths for tumor assessment, such as large data sources because radiologic images are available in almost all cancer patients, measurement of heterogeneity of entire tumor, and longitudinal use in treatment monitoring [14, 38]. In our results, radiomics features were not correlated with genomics data especially in Kaplan-Meier survival curves, indicating that radiomics may provide additional information on cancer characterization that are different from genomics. The integration of radiomics and genomics data could be valuable and enable more precise survival prediction. However, the prognostic role of radiomics and other omics features is still controversial, for example, few studies demonstrated that *VHL* mutation could predict patient survival [9], and many genes and pathways still lack related evidence. Moreover, the correlation between radiomics and other omics features is complex. Therefore, further investigation to reveal the causal relationship between these features is needed.

There were several limitations in this study, including the retrospective study design and limited sample size. We randomly divided TCGA patients into training and validation sets to verify the robustness of predictive models in validation set. However, the quality of images from public dataset showed great differences, which may influence the radiomics analysis. Thus, we further estimated the generalizability and reliability of radiomics model on an external validation set. However, the genomics, transcriptomics and proteomics data were lacked in external validation set. It was necessary to assess our integrative models in other datasets and populations. In addition, small patient populations with *BAP1* or *SETD2* mutations might cause potential bias, which was a common problem in other radiogenomics studies. Therefore, before clinical application, the findings based on the limited dataset need to be prospectively validated on multi-center and large-scale studies.

## CONCLUSIONS

Radiomics features of contrast-enhanced CT had promising potential in predicting mutation status, molecular subtypes and overall survival of patients with ccRCC. Moreover, integrative models of radiomics, genomics, transcriptomics and proteomics could improve prognostic prediction compared with radiomics or other omics alone, which may help clinicians to perform better risk stratification and decision-making for ccRCC patients.

## MATERIALS AND METHODS

### Study design and data sources

**Figure 6:**
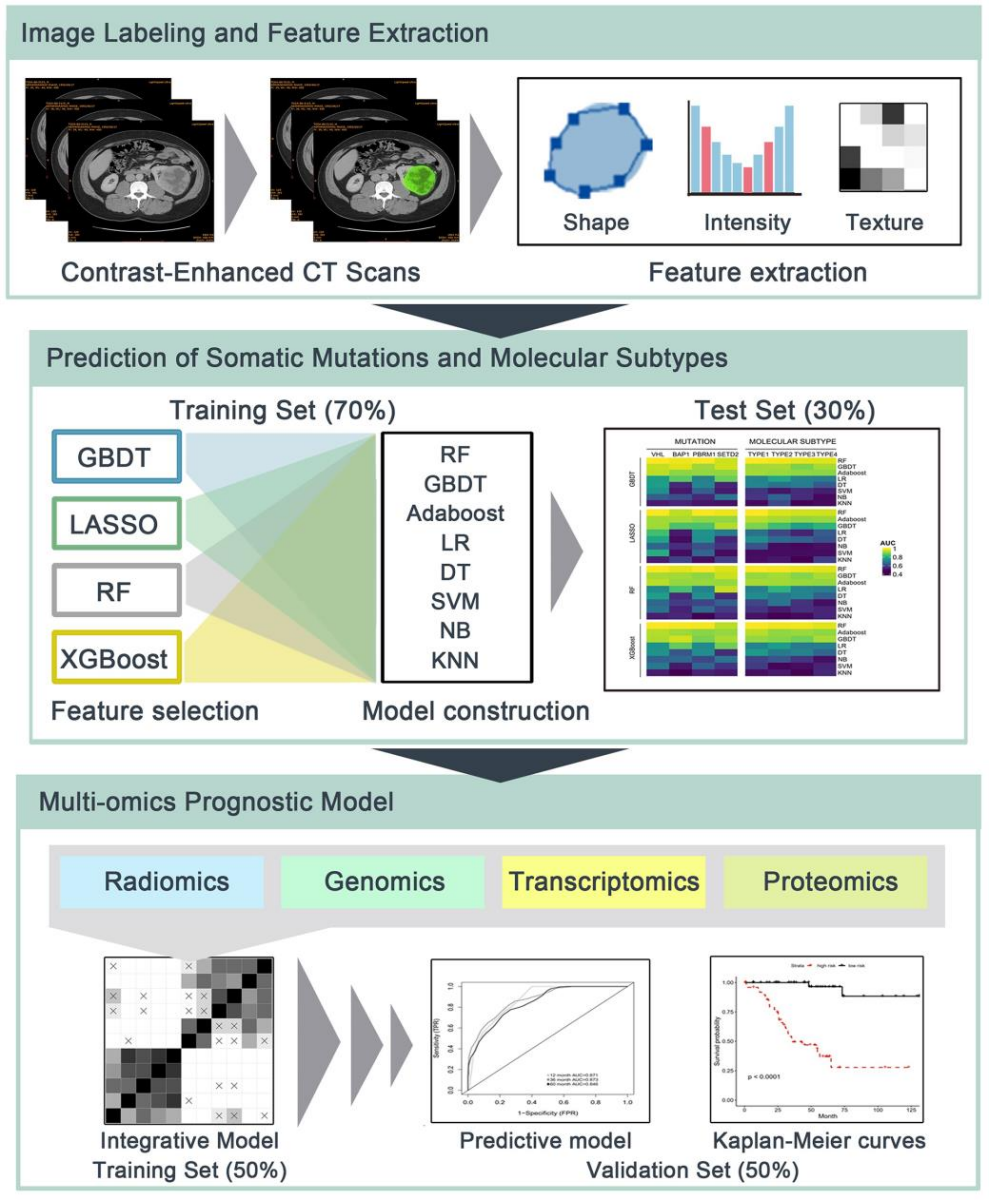
**The flowchart of radiomics analysis and omics integration.** (1) Manual delineation of tumor region of interest (ROI) of contrast-enhanced CT. Shape-based, first-order and second-order radiomics features of ROIs were then calculated. (2) Prediction of somatic mutations and molecular subtypes using radiomics features and multiple machine learning algorithms in independent training/test sets. (3) Radiomics, genomics, transcriptomics and proteomics were integrated to build predictive models for overall survival in training set, and their prognostic values were estimated using validation set.

In this retrospective study, radiomics features were applied to predict somatic mutations and molecular subtypes, and to form integrative models for survival prediction in ccRCC patients (Figure 6). One cohort of ccRCC patients with available CT images was downloaded from The Cancer Imaging Archive (TCIA) data portal (http://www.cancerimagingarchive.net/). The matched clinical data, genomics, transcriptomics and proteomics profiles were obtained from The Cancer Genome Atlas (TCGA) (https://portal.gdc.cancer.gov/) and The Cancer Proteome Atlas (TCPA) (http://tcpaportal.org/tcpa/). The inclusion criteria were pathologically confirmed ccRCC patients with preoperative contrast-enhanced CT for better tumor visualization. Patients without preoperative CT (n=16) or without contrast-enhanced CT (n=14) were excluded, thus 207 patients were eligible for radiomics analysis.

Another ccRCC cohort was diagnosed in the West China Hospital from January 2011 to December 2018. We excluded the patients without preoperative contrast-enhanced CT (n=33) or lost to follow-up (n=21). Finally, a total of 175 patients with ccRCC were enrolled in this study. All these patients were followed up until death or last follow-up of December 2020. The Ethics committee of West China Hospital approved this study. All personal information was de-identified, thus the patient informed consents were waived.

### Image labeling and feature extraction

Before feature extraction, an expert oncologist (X.M., 10 years of experience) that blinded to patients’ information manually draw the region of interest (ROI) of the whole tumor. We used the 3D Slicer software 4.10.2 (http://www.slicer.org) for both ROI labeling and feature extraction. Radiomics features can be divided into shape-based, first-order, second-order and higher-order statistical metrics. Shape-based features measure the three-dimensional shape and size of tumor, including surface area, volume, sphericity and others. First-order features are generally based on histogram-related methods, which represent the value distribution of individual voxels without considering the spatial relationship. For example, skewness describes the degree of asymmetry of histogram distribution, and kurtosis refers to the peakedness of histogram. Second-order features evaluate the spatial relationship between voxels with similar contrast levels to reveal the intratumoral heterogeneity, including gray-level co-occurrence matrix (GLCM), gray-level dependence matrix (GLDM), gray-level run length matrix (GLRLM), gray-level size zone matrix (GLSZM), neighborhood gray tone difference matrix (NGTDM) and so on. Finally, there were 107 quantitative features for each patient, which contained 14 shape-based features, 18 histogram-based features, 24 GLCM features, 14 GLDM features, 16 GLRLM features, 16 GLSZM features and 5 NGTDM features.

### Statistical analysis

1. Prediction of mutations and subtypes: We divided the TCGA cohort into training and test sets with the ratio of 7:3. Considering the risk of over-fitting due to data complexity, we reduced the dimensionality of radiomics features by machine learning algorithms including gradient boosting decision tree (GBDT) [39], least absolute shrinkage and selection operator (LASSO) [40], random forest (RF) [41] and extreme gradient boosting (XGBoost) [42]. Afterwards, eight machine learning algorithms, namely RF, GBDT, adaptive boosting (AdaBoost) [43], logistic regression (LR) [43], decision tree (DT) [44], support vector machine (SVM) [45], naive Bayesian (NB) [46] and K-nearest neighbor (KNN) [47] were utilized to establish binary classifiers on remained radiomics features to predict somatic mutations (*VHL*, *BAP1*, *PBRM1* and *SETD2*) and mRNA-based molecular subtypes (m1-m4). The reason for using multiple algorithms was to indicate the feasibility of this method in different algorithms. The 5-fold cross-validation was used to assess classifiers in the training set. Finally, the performances of trained classifiers were independently validated by the area under the receiver operating characteristic (ROC) curve (AUC) in the test set.

2. Survival analysis: According to median value of radiomics features, TCGA patients were divided into high-value and low-value groups. Compared with progression-free survival, the evaluation of overall survival (OS) is more objective and less affected by artificial factors. We chose the OS as the endpoint to better explore the prognostic performance of features. Univariate Cox regression analysis calculated the hazard ratio (HR) and 95% confidence interval (CI) for OS between two groups. Then we used the multivariate Cox regression with LASSO to shrink the regression coefficients of uncorrelated features to zero, and obtained features with non-zero coefficients. Kaplan-Meier survival curve and log-rank test were analyzed. The p<0.05 was considered as statistically significant.

3. Model features pre-selection: To comprehensively present the prognostic value of each omics, we estimated prognostic models based on one omics data (radiomics, genomics, transcriptomics, proteomics) and combinations (radiomics+genomics, radiomics+transcriptomics, radiomics+proteomics, multi-omics). The TCGA cohort was randomly divided into training (n=104) or validation (n=103) sets. To reduce the dimensionality of genomics data, 100 most common mutations of training set were used for building models. For transcriptomics, we first assigned the training set into short-term (deceased, 12 months≥OS≥1 month) and long-term (OS≥60 months) survivors. Differently expressed genes (DEGs) between two groups were further involved in models. In addition, we applied the Gene Set Enrichment Analysis (GSEA) to show enriched Kyoto Encyclopedia of Genes and Genomes (KEGG) pathways. Gene sets were significant if the p<0.05 or false discovery rate (FDR)<0.25.

4. Prognostic model construction and validation: In the training set, random forest (RF) was used to establish prognostic models, because of its excellent performance of handling numerous inputs and selecting relevant features. The 1000 decision trees of RF and 5-fold cross-validation were used. In the validation set, we verified the robustness of these models using the time-dependent ROC curve and compared AUC values through the built-in comparison function of timeROC package. Patients in validation set were then separated into high-risk and low-risk groups according to median value of risk score estimated by models. In addition, decision curve analysis (DCA) was applied to compare the net benefits on threshold probabilities of models [48]. Statistical analyses were conducted using R version 3.6.1.

## Supplementary Material

Supplementary Figure

Supplementary Table 1

Supplementary Table 2

Supplementary Table 3

Supplementary Table 4

Supplementary Table 5

Supplementary Table 6

## References

[1] Srigley JR, Delahunt B, Eble JN, Egevad L, Epstein JI, Grignon D, Hes O, Moch H, Montironi R, Tickoo SK, Zhou M, Argani P, and ISUP Renal Tumor Panel. The international society of urological pathology (ISUP) vancouver classification of renal neoplasia. Am J Surg Pathol. 2013; 37:1469–89. 10.1097/PAS.0b013e318299f2d1 24025519

[2] Bray F, Ferlay J, Soerjomataram I, Siegel RL, Torre LA, Jemal A. Global cancer statistics 2018: GLOBOCAN estimates of incidence and mortality worldwide for 36 cancers in 185 countries. CA Cancer J Clin. 2018; 68:394–424. 10.3322/caac.21492 30207593

[3] Shuch B, Amin A, Armstrong AJ, Eble JN, Ficarra V, Lopez-Beltran A, Martignoni G, Rini BI, Kutikov A. Understanding pathologic variants of renal cell carcinoma: distilling therapeutic opportunities from biologic complexity. Eur Urol. 2015; 67:85–97. 10.1016/j.eururo.2014.04.029 24857407

[4] Leibovich BC, Lohse CM, Crispen PL, Boorjian SA, Thompson RH, Blute ML, Cheville JC. Histological subtype is an independent predictor of outcome for patients with renal cell carcinoma. J Urol. 2010; 183:1309–15. 10.1016/j.juro.2009.12.035 20171681

[5] Tang PA, Vickers MM, Heng DY. Clinical and molecular prognostic factors in renal cell carcinoma: what we know so far. Hematol Oncol Clin North Am. 2011; 25:871–91. 10.1016/j.hoc.2011.04.003 21763972

[6] Cancer Genome Atlas Research Network. Comprehensive molecular characterization of clear cell renal cell carcinoma. Nature. 2013; 499:43–49. 10.1038/nature12222 23792563 PMC3771322

[7] Rechsteiner MP, von Teichman A, Nowicka A, Sulser T, Schraml P, Moch H. VHL gene mutations and their effects on hypoxia inducible factor HIFα: identification of potential driver and passenger mutations. Cancer Res. 2011; 71:5500–11. 10.1158/0008-5472.CAN-11-0757 21715564

[8] Rini BI, Escudier B, Tomczak P, Kaprin A, Szczylik C, Hutson TE, Michaelson MD, Gorbunova VA, Gore ME, Rusakov IG, Negrier S, Ou YC, Castellano D, et al. Comparative effectiveness of axitinib versus sorafenib in advanced renal cell carcinoma (AXIS): a randomised phase 3 trial. Lancet. 2011; 378:1931–39. 10.1016/S0140-6736(11)61613-9 22056247

[9] Kim HS, Kim JH, Jang HJ, Han B, Zang DY. Clinicopathologic significance of *VHL* gene alteration in clear-cell renal cell carcinoma: an updated meta-analysis and review. Int J Mol Sci. 2018; 19:2529. 10.3390/ijms19092529 30149673 PMC6165550

[10] Wang Z, Peng S, Guo L, Xie H, Wang A, Shang Z, Niu Y. Prognostic and clinicopathological value of PBRM1 expression in renal cell carcinoma. Clin Chim Acta. 2018; 486:9–17. 10.1016/j.cca.2018.07.014 30006290

[11] Miao D, Margolis CA, Gao W, Voss MH, Li W, Martini DJ, Norton C, Bossé D, Wankowicz SM, Cullen D, Horak C, Wind-Rotolo M, Tracy A, et al. Genomic correlates of response to immune checkpoint therapies in clear cell renal cell carcinoma. Science. 2018; 359:801–06. 10.1126/science.aan5951 29301960 PMC6035749

[12] Hakimi AA, Ostrovnaya I, Reva B, Schultz N, Chen YB, Gonen M, Liu H, Takeda S, Voss MH, Tickoo SK, Reuter VE, Russo P, Cheng EH, et al, and ccRCC Cancer Genome Atlas (KIRC TCGA) Research Network investigators. Adverse outcomes in clear cell renal cell carcinoma with mutations of 3p21 epigenetic regulators BAP1 and SETD2: a report by MSKCC and the KIRC TCGA research network. Clin Cancer Res. 2013; 19:3259–67. 10.1158/1078-0432.CCR-12-3886 23620406 PMC3708609

[13] Hsieh JJ, Chen D, Wang PI, Marker M, Redzematovic A, Chen YB, Selcuklu SD, Weinhold N, Bouvier N, Huberman KH, Bhanot U, Chevinsky MS, Patel P, et al. Genomic biomarkers of a randomized trial comparing first-line everolimus and sunitinib in patients with metastatic renal cell carcinoma. Eur Urol. 2017; 71:405–14. 10.1016/j.eururo.2016.10.007 27751729 PMC5431298

[14] Gillies RJ, Kinahan PE, Hricak H. Radiomics: images are more than pictures, they are data. Radiology. 2016; 278:563–77. 10.1148/radiol.2015151169 26579733 PMC4734157

[15] Meng Y, Sun J, Qu N, Zhang G, Yu T, Piao H. Application of radiomics for personalized treatment of cancer patients. Cancer Manag Res. 2019; 11:10851–58. 10.2147/CMAR.S232473 31920394 PMC6941598

[16] Bodalal Z, Trebeschi S, Nguyen-Kim TD, Schats W, Beets-Tan R. Radiogenomics: bridging imaging and genomics. Abdom Radiol (NY). 2019; 44:1960–84. 10.1007/s00261-019-02028-w 31049614

[17] Grossmann P, Stringfield O, El-Hachem N, Bui MM, Rios Velazquez E, Parmar C, Leijenaar RT, Haibe-Kains B, Lambin P, Gillies RJ, Aerts HJ. Defining the biological basis of radiomic phenotypes in lung cancer. Elife. 2017; 6:e23421. 10.7554/eLife.23421 28731408 PMC5590809

[18] Yang L, Dong D, Fang M, Zhu Y, Zang Y, Liu Z, Zhang H, Ying J, Zhao X, Tian J. Can CT-based radiomics signature predict KRAS/NRAS/BRAF mutations in colorectal cancer? Eur Radiol. 2018; 28:2058–67. 10.1007/s00330-017-5146-8 29335867

[19] Saha A, Harowicz MR, Grimm LJ, Kim CE, Ghate SV, Walsh R, Mazurowski MA. A machine learning approach to radiogenomics of breast cancer: a study of 922 subjects and 529 DCE-MRI features. Br J Cancer. 2018; 119:508–16. 10.1038/s41416-018-0185-8 30033447 PMC6134102

[20] Li ZC, Bai H, Sun Q, Li Q, Liu L, Zou Y, Chen Y, Liang C, Zheng H. Multiregional radiomics features from multiparametric MRI for prediction of MGMT methylation status in glioblastoma multiforme: a multicentre study. Eur Radiol. 2018; 28:3640–50. 10.1007/s00330-017-5302-1 29564594

[21] Chaddad A, Daniel P, Sabri S, Desrosiers C, Abdulkarim B. Integration of radiomic and multi-omic analyses predicts survival of newly diagnosed IDH1 wild-type glioblastoma. Cancers (Basel). 2019; 11:1148. 10.3390/cancers11081148 31405148 PMC6721570

[22] Wijethilake N, Islam M, Ren H. Radiogenomics model for overall survival prediction of glioblastoma. Med Biol Eng Comput. 2020; 58:1767–77. 10.1007/s11517-020-02179-9 32488372

[23] Badic B, Hatt M, Durand S, Jossic-Corcos CL, Simon B, Visvikis D, Corcos L. Radiogenomics-based cancer prognosis in colorectal cancer. Sci Rep. 2019; 9:9743. 10.1038/s41598-019-46286-6 31278324 PMC6611779

[24] Chen X, Zhou Z, Hannan R, Thomas K, Pedrosa I, Kapur P, Brugarolas J, Mou X, Wang J. Reliable gene mutation prediction in clear cell renal cell carcinoma through multi-classifier multi-objective radiogenomics model. Phys Med Biol. 2018; 63:215008. 10.1088/1361-6560/aae5cd 30277889 PMC6240911

[25] Feng Z, Zhang L, Qi Z, Shen Q, Hu Z, Chen F. Identifying BAP1 mutations in clear-cell renal cell carcinoma by CT radiomics: preliminary findings. Front Oncol. 2020; 10:279. 10.3389/fonc.2020.00279 32185138 PMC7058626

[26] Kamarudin AN, Cox T, Kolamunnage-Dona R. Time-dependent ROC curve analysis in medical research: current methods and applications. BMC Med Res Methodol. 2017; 17:53. 10.1186/s12874-017-0332-6 28388943 PMC5384160

[27] Saxena M, Yeretssian G. NOD-like receptors: master regulators of inflammation and cancer. Front Immunol. 2014; 5:327. 10.3389/fimmu.2014.00327 25071785 PMC4095565

[28] Liu Z, Wang S, Dong D, Wei J, Fang C, Zhou X, Sun K, Li L, Li B, Wang M, Tian J. The applications of radiomics in precision diagnosis and treatment of oncology: opportunities and challenges. Theranostics. 2019; 9:1303–22. 10.7150/thno.30309 30867832 PMC6401507

[29] Hanahan D, Weinberg RA. Hallmarks of cancer: the next generation. Cell. 2011; 144:646–74. 10.1016/j.cell.2011.02.013 21376230

[30] Berger MF, Mardis ER. The emerging clinical relevance of genomics in cancer medicine. Nat Rev Clin Oncol. 2018; 15:353–65. 10.1038/s41571-018-0002-6 29599476 PMC6658089

[31] Kocak B, Durmaz ES, Ates E, Ulusan MB. Radiogenomics in clear cell renal cell carcinoma: machine learning-based high-dimensional quantitative CT texture analysis in predicting PBRM1 mutation status. AJR Am J Roentgenol. 2019; 212:W55–63. 10.2214/AJR.18.20443 30601030

[32] Kocak B, Durmaz ES, Kaya OK, Kilickesmez O. Machine learning-based unenhanced CT texture analysis for predicting BAP1 mutation status of clear cell renal cell carcinomas. Acta Radiol. 2020; 61:856–64. 10.1177/0284185119881742 31635476

[33] Brannon AR, Reddy A, Seiler M, Arreola A, Moore DT, Pruthi RS, Wallen EM, Nielsen ME, Liu H, Nathanson KL, Ljungberg B, Zhao H, Brooks JD, et al. Molecular stratification of clear cell renal cell carcinoma by consensus clustering reveals distinct subtypes and survival patterns. Genes Cancer. 2010; 1:152–63. 10.1177/1947601909359929 20871783 PMC2943630

[34] Suarez-Ibarrola R, Basulto-Martinez M, Heinze A, Gratzke C, Miernik A. Radiomics applications in renal tumor assessment: a comprehensive review of the literature. Cancers (Basel). 2020; 12:1387. 10.3390/cancers12061387 32481542 PMC7352711

[35] Nardone V, Tini P, Nioche C, Mazzei MA, Carfagno T, Battaglia G, Pastina P, Grassi R, Sebaste L, Pirtoli L. Texture analysis as a predictor of radiation-induced xerostomia in head and neck patients undergoing IMRT. Radiol Med. 2018; 123:415–23. 10.1007/s11547-017-0850-7 29368244

[36] Tarsitano A, Ricotta F, Cercenelli L, Bortolani B, Battaglia S, Lucchi E, Marchetti C, Marcelli E. Pretreatment tumor volume and tumor sphericity as prognostic factors in patients with oral cavity squamous cell carcinoma. J Craniomaxillofac Surg. 2019; 47:510–15. 10.1016/j.jcms.2018.12.019 30642733

[37] Guezennec C, Robin P, Orlhac F, Bourhis D, Delcroix O, Gobel Y, Rousset J, Schick U, Salaün PY, Abgral R. Prognostic value of textural indices extracted from pretherapeutic 18-F FDG-PET/CT in head and neck squamous cell carcinoma. Head Neck. 2019; 41:495–502. 10.1002/hed.25433 30549149

[38] Jansen RW, van Amstel P, Martens RM, Kooi IE, Wesseling P, de Langen AJ, Menke-Van der Houven van Oordt CW, Jansen BH, Moll AC, Dorsman JC, Castelijns JA, de Graaf P, de Jong MC. Non-invasive tumor genotyping using radiogenomic biomarkers, a systematic review and oncology-wide pathway analysis. Oncotarget. 2018; 9:20134–55. 10.18632/oncotarget.24893 29732009 PMC5929452

[39] Friedman JH. Greedy Function Approximation: A Gradient Boosting Machine. Ann Stat. 2001; 29:1189–32. 10.1214/aos/1013203451

[40] Tibshirani R. The lasso method for variable selection in the cox model. Stat Med. 1997; 16:385–95. 10.1002/(sici)1097-0258(19970228)16:4<385::aid-sim380>3.0.co;2-3 9044528

[41] Breiman L. Random Forests. Mach Learn. 2001; 45:5–32. 10.1023/A:1010933404324

[42] Chen T, Guestrin C. XGBoost: a Scalable Tree Boosting System. Proceedings of the 22nd ACM SIGKDD International Conference on Knowledge Discovery and Data Mining. 2016; 785–94. 10.1145/2939672.2939785

[43] Collins M, Schapire RE, Singer Y. Logistic Regression, AdaBoost and Bregman Distances. Mach Learn. 2002; 48:253–85. 10.1023/A:1013912006537

[44] Safavian SR, Landgrebe D. A survey of decision tree classifier methodology. IEEE Trans Syst Man Cybern. 1991; 21:660–74. 10.1109/21.97458

[45] Cortes C, Vapnik VN. Support Vector Networks. Mach Learn. 1995; 20:273–97. 10.1007/BF00994018

[46] Friedman N, Geiger D, Goldszmidt M. Bayesian Network Classifiers. Mach Learn. 1997; 29:131–63. 10.1023/A:1007465528199

[47] Keller JM, Gray MR, Givens JA. A fuzzy K-nearest neighbor algorithm. IEEE Trans Syst Man Cybern. 1985; SMC-15:580–85. 10.1109/TSMC.1985.6313426

[48] Vickers AJ, Cronin AM, Elkin EB, Gonen M. Extensions to decision curve analysis, a novel method for evaluating diagnostic tests, prediction models and molecular markers. BMC Med Inform Decis Mak. 2008; 8:53. 10.1186/1472-6947-8-53 19036144 PMC2611975

