# Supplementary material for: Integrative radiogenomics analysis for predicting molecular features and survival in clear cell renal cell carcinoma
Source: Aging (Albany NY). 2021 Mar 26;13(7):9960–75. doi: 10.18632/aging.202752 (PMC8064160; doi:10.18632/aging.202752)
Supplement: Supplementary Figure [file aging-13-202752-s001.pdf]

SUPPLEMENTARY FIGURE

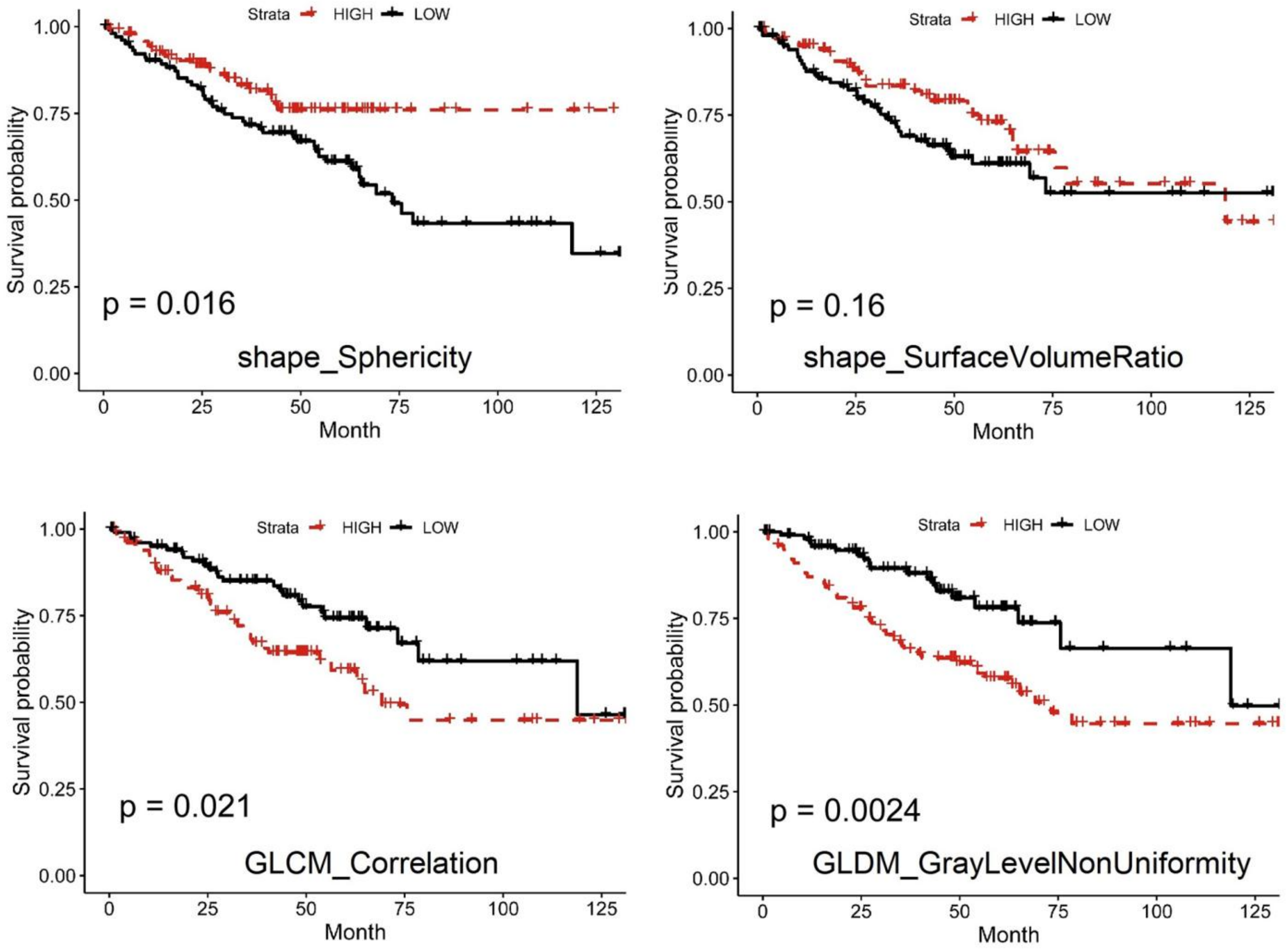

Supplementary Figure 1. Kaplan-Meier curves of groups with high-value and low-value "sphericity", "surface-to-volume ratio", "GLCM_correlation" and "GLDM_gray-level non-uniformity".
