# Supplementary material for: Integrative radiogenomics analysis for predicting molecular features and survival in clear cell renal cell carcinoma
Source: Aging (Albany NY). 2021 Mar 26;13(7):9960–75. doi: 10.18632/aging.202752 (PMC8064160; doi:10.18632/aging.202752)
Supplement: Supplementary Table 1 [file aging-13-202752-s002.pdf]

Supplementary Table 1. Performance of machine learning algorithms in predicting somatic mutations and molecular subtypes.

Algorithm		Somatic Mutation				Molecular Subtype			
		VHL	BAP1	PBRM1	SETD2	m1	m2	m3	m4
GBDT	RF	0.977	0.967	0.976	0.941	0.975	0.968	0.960	0.955
	GBDT	0.936	0.955	0.907	0.945	0.911	0.915	0.884	0.914
	AdaBoost	0.929	0.897	0.903	0.899	0.913	0.906	0.899	0.896
	LR	0.765	0.858	0.714	0.871	0.717	0.782	0.712	0.700
	DT	0.768	0.557	0.719	0.554	0.724	0.681	0.703	0.639
	SVM	0.744	0.535	0.648	0.538	0.576	0.527	0.562	0.544
	NB	0.646	0.617	0.560	0.688	0.578	0.655	0.564	0.514
	KNN	0.537	0.552	0.530	0.526	0.516	0.608	0.504	0.517
LASSO	RF	0.972	0.926	0.970	0.961	0.973	0.947	0.941	0.940
	AdaBoost	0.908	0.909	0.895	0.894	0.924	0.909	0.895	0.900
	GBDT	0.905	0.836	0.834	0.919	0.879	0.825	0.764	0.803
	LR	0.827	0.520	0.733	0.684	0.669	0.589	0.532	0.626
	DT	0.772	0.500	0.726	0.534	0.728	0.653	0.639	0.607
	NB	0.642	0.550	0.604	0.560	0.611	0.500	0.495	0.505
	SVM	0.789	0.500	0.647	0.500	0.500	0.500	0.500	0.500
	KNN	0.523	0.582	0.504	0.574	0.502	0.518	0.486	0.577
RF	RF	0.971	0.955	0.972	0.949	0.973	0.968	0.961	0.953
	GBDT	0.910	0.907	0.894	0.936	0.898	0.909	0.887	0.915
	AdaBoost	0.925	0.898	0.900	0.899	0.912	0.908	0.902	0.898
	LR	0.722	0.835	0.710	0.932	0.704	0.803	0.712	0.700
	DT	0.764	0.556	0.734	0.554	0.726	0.681	0.712	0.639
	NB	0.655	0.579	0.594	0.642	0.597	0.683	0.564	0.514
	SVM	0.676	0.540	0.658	0.578	0.568	0.528	0.562	0.544
	KNN	0.536	0.551	0.496	0.544	0.519	0.496	0.507	0.546
XGBoost	RF	0.975	0.970	0.970	0.927	0.974	0.965	0.967	0.955
	AdaBoost	0.922	0.904	0.893	0.900	0.926	0.897	0.904	0.896
	GBDT	0.917	0.944	0.880	0.915	0.900	0.891	0.873	0.914
	LR	0.737	0.862	0.724	0.872	0.714	0.760	0.693	0.700
	DT	0.746	0.623	0.733	0.554	0.758	0.690	0.709	0.654
	NB	0.643	0.657	0.596	0.681	0.590	0.621	0.567	0.514
	SVM	0.688	0.528	0.648	0.568	0.579	0.502	0.511	0.544
	KNN	0.532	0.495	0.539	0.526	0.512	0.529	0.498	0.548

Abbreviations: GBDT: gradient boosting decision tree; LASSO: least absolute shrinkage and selection operator; RF: random forest; XGBoost: extreme gradient boosting; AdaBoost: adaptive boosting; LR: logistic regression; DT: decision tree; SVM: support vector machine; NB: naive Bayesian; KNN: K-nearest neighbor.
