# Supplementary material for: Integrative radiogenomics analysis for predicting molecular features and survival in clear cell renal cell carcinoma
Source: Aging (Albany NY). 2021 Mar 26;13(7):9960–75. doi: 10.18632/aging.202752 (PMC8064160; doi:10.18632/aging.202752)
Supplement: Supplementary Table 4 [file aging-13-202752-s005.pdf]

Supplementary Table 4. Comparison of prognostic performance of models according to AUC.

p value	Radiomics	Radiomics+ genomics	Radiomics+transcriptomics	Radiomics+ proteomics	Multi-omics
Radiomics					
1-year AUC	-	0.012	0.008	0.003	1.04×10^{-5}
2-year AUC	-	0.026	0.0007	0.009	0.003
3-year AUC	-	0.016	0.002	0.062	0.005
Genomics					
1-year AUC	0.247	0.026	-	-	0.0002
2-year AUC	0.069	0.005	-	-	3.40×10^{-5}
3-year AUC	0.030	0.008	-		0.0002
Transcriptomics					
1-year AUC	0.159	-	0.007		0.001
2-year AUC	0.246	-	0.011	-	0.001
3-year AUC	0.674	-	0.003	-	0.007
Proteomics					
1-year AUC	0.053	-	-	0.006	0.008
2-year AUC	0.293	-	-	0.011	0.002
3-year AUC	0.868	-	-	0.029	0.009
